# Vogt‐Koyanagi‐Harada disease‐like uveitis induced by nivolumab in metastatic renal cell carcinoma

**DOI:** 10.1002/iju5.12801

**Published:** 2024-10-21

**Authors:** Hitomi Imai, Tomoyuki Koguchi, Yuki Harigane, Kei Yaginuma, Satoru Meguro, Seiji Hoshi, Junya Hata, Hidenori Akaihata, Soichiro Ogawa, Yoshiyuki Kojima

**Affiliations:** ^1^ Department of Urology Fukushima Medical University School of Medicine Fukushima Japan

**Keywords:** HLA‐DR4, immune‐related adverse events, metastatic renal cell carcinoma, nivolumab, Vogt‐Koyanagi‐Harada disease‐like uveitis

## Abstract

**Introduction:**

Nivolumab can cause various immune‐related adverse events; it rarely induces Vogt‐Koyanagi‐Harada‐disease‐like uveitis. Vogt‐Koyanagi‐Harada‐disease is reported to be closely associated with human leukocyte antigen‐DR4.

**Case presentation:**

A 68‐year‐old man with metastatic renal cancer underwent nephrectomy. Computed tomography showed multiple lung tumors, raising suspicion of lung metastases. However, one lung hilar mass was suspected to be primary lung cancer, leading to a lobectomy, which subsequently revealed lung metastases of renal cancer. The patient underwent nivolumab treatment but developed Vogt‐Koyanagi‐Harada‐disease‐like uveitis as an immune‐related adverse event. Human leukocyte antigen‐DR4 alleles were identified, and the uveitis improved with topical steroids. He maintained partial response of lung metastases after nivolumab resumption. Immunohistochemical staining revealed significantly higher human leukocyte antigen‐DR4 expression in lung metastasis than primary renal cancer.

**Conclusion:**

Despite inducing Vogt‐Koyanagi‐Harada‐disease‐like uveitis, nivolumab controlled cancer progression effectively. Immunohistochemical staining results suggest the potential involvement of human leukocyte antigen‐DR4 expression in both the onset of Vogt‐Koyanagi‐Harada‐disease‐like uveitis and nivolumab efficacy.

**Abbreviations & Acronyms**
APC: antigen‐presenting cell
ARMC9: armadillo repeat containing 9
CT: Computed tomography
HLA: human leukocyte antigen
ICI: immune checkpoint inhibitor
irAE: immune‐related adverse event
mRCC: metastatic renal cell carcinoma
PD‐1: programmed cell death‐1
PD‐L1: programmed death‐ligand 1
PR: partial response
RCC: renal cell carcinoma
VKH: Vogt‐Koyanagi‐Harada‐disease

**Keynote message**

A patient with metastatic renal cell carcinoma (mRCC) developed Vogt‐Koyanagi‐Harada (VKH)‐like uveitis as an immune‐related adverse event of nivolumab. The uveitis was managed with topical steroids, and the mRCC was controlled by nivolumab treatment. HLA‐DR4, associated with VKH and immune response, might influence both VKH‐like uveitis and nivolumab efficacy.

## Introduction

Nivolumab, an anti‐PD‐1 inhibitor, is effective for treating mRCC.^1^ However, it causes various irAEs.^2^ Previous reports have shown that VKH‐like uveitis is a rare irAEs, occurring in an estimated 1% of patients receiving a combination of ipilimumab and nivolumab.^2^ VKH‐like uveitis is an autoimmune disease in which autoreactive T cells attack melanocytes in the retina, resulting in vision loss.^3^ Early diagnosis and treatment of VKH‐like uveitis as an irAE is important. We here report a case of nivolumab‐induced VKH‐like uveitis in a patient with mRCC. The VKH‐like uveitis was managed with topical steroid therapy, allowing continued treatment of mRCC with nivolumab.

## Case report

**Fig. 1:**
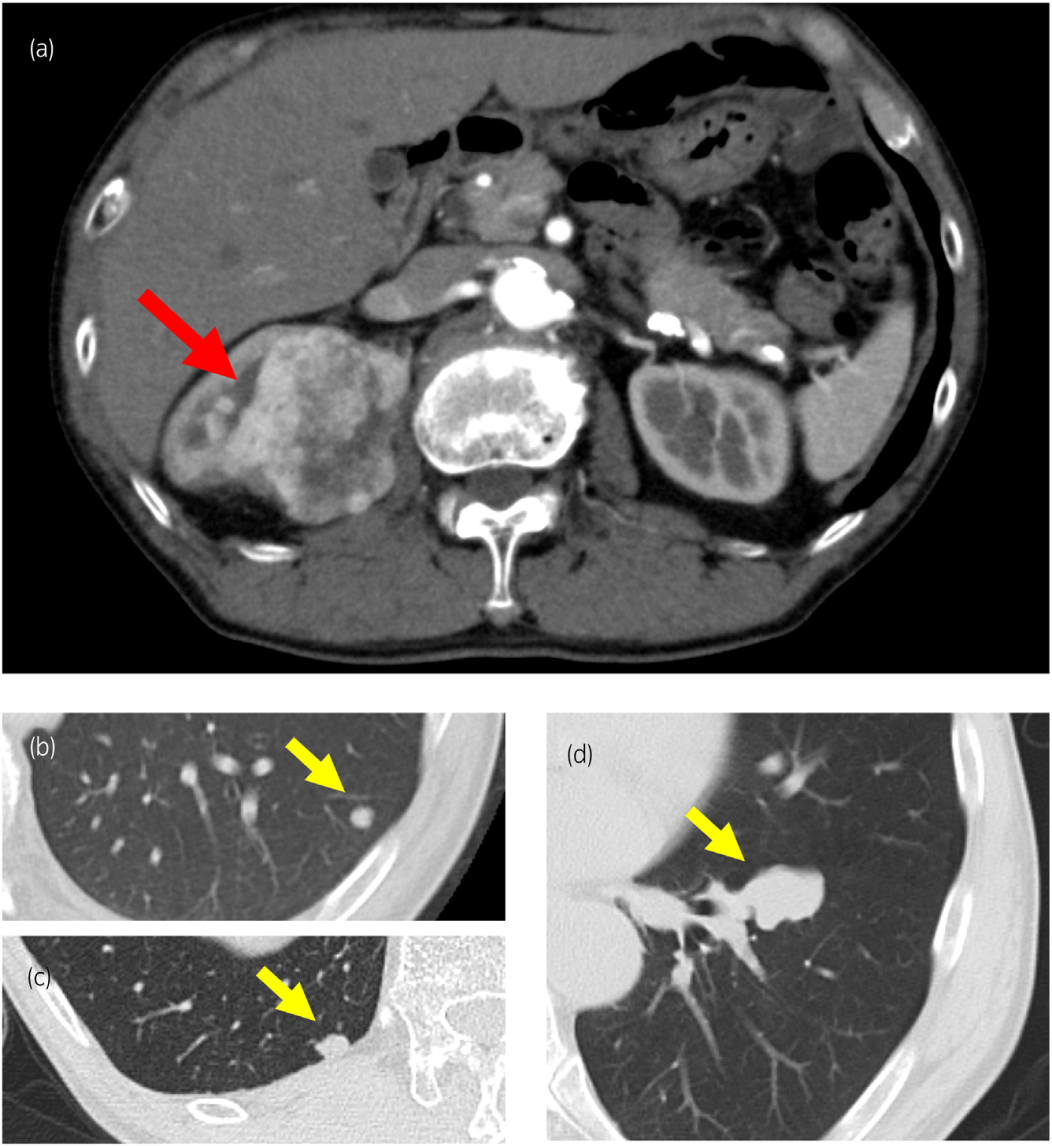
(a) Abdominal CT scan revealed a 65 mm × 60 mm tumor (red arrow) in the right kidney. (b–d) Thoracic CT scan of the chest showed multiple lung tumors (yellow arrows). Among them, the 35 mm hilar mass (d) was indistinguishable as either primary lung cancer or lung metastasis of RCC.

**Fig. 2:**
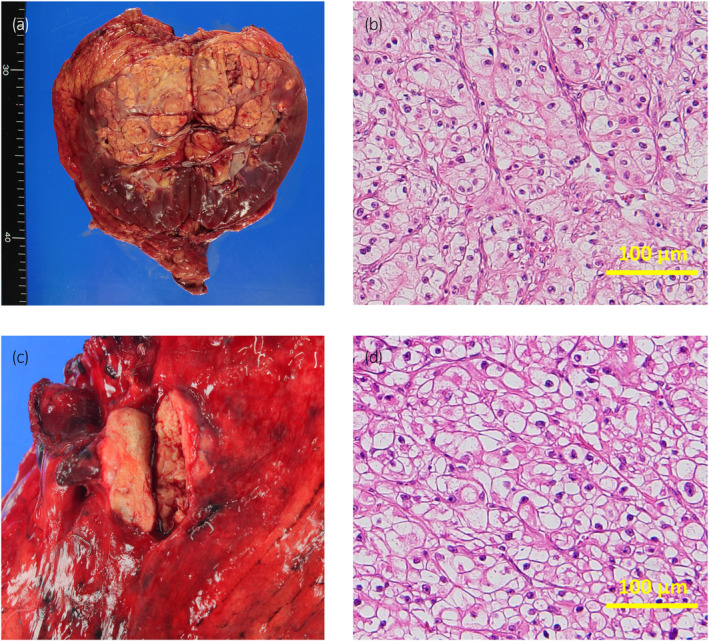
(a) The surgical specimen from radical nephrectomy shows a tumor with a solid and yellow appearance on the cut surface. (b) Microphotograph of the renal tumor with hematoxylin and eosin staining reveals clear or granular eosinophilic cytoplasm. Scale bar: 100 μm. (c) The surgical specimen of the thoracoscopic lung lobectomy revealed a tumor with a solid and yellow appearance similar to the renal tumor on the cut surface. (d) The microphotograph of the lung metastasis with hematoxylin and eosin staining also showed clear or granular eosinophilic cytoplasm, resembling that of the renal tumor. Scale bar: 100 μm.

**Fig. 3:**
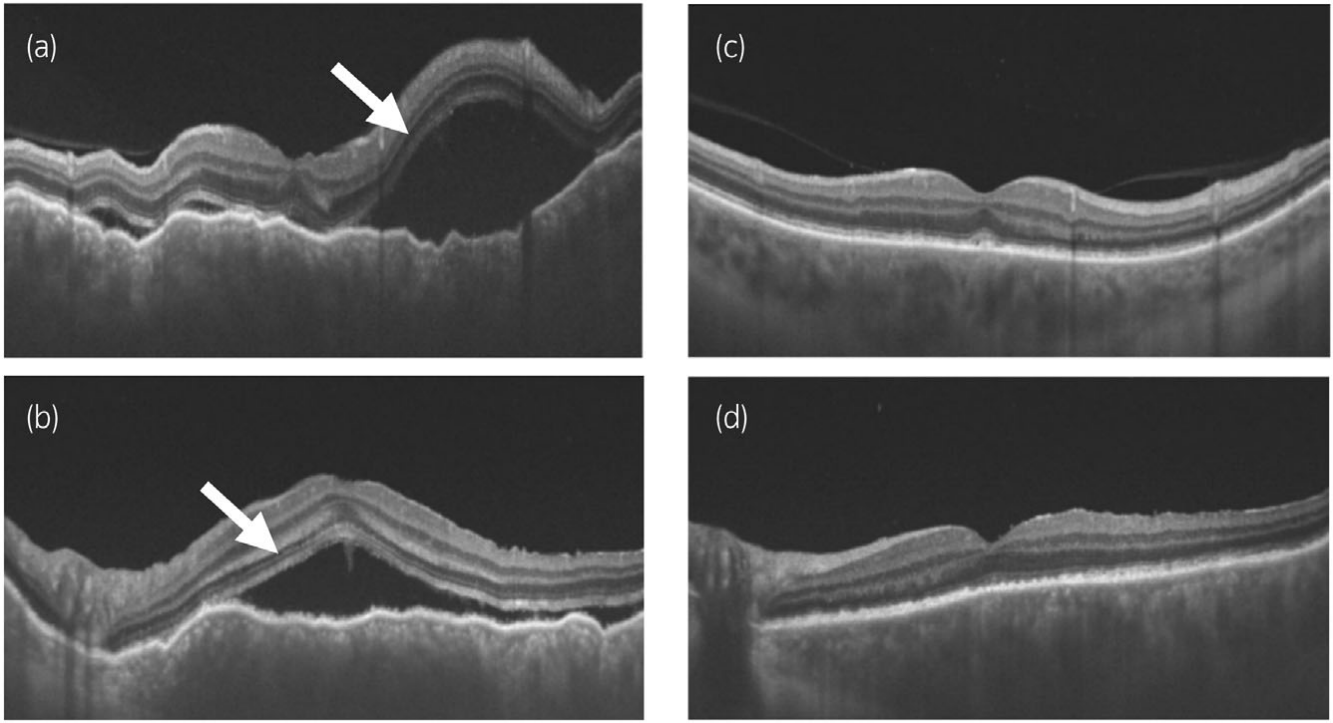
Optical coherence tomography images show that subretinal fluid (white arrows) was present in the right (a) and left (b) eyes before topical steroid therapy for Vogt‐Koyanagi‐Harada disease‐like uveitis. 60 days after topical steroid treatment, the right (c) and left (d) subretinal fluid were resolved.

A 68‐year‐old male patient was referred to our hospital for detailed evaluation of a right renal tumor. CT showed a right renal tumor measuring 65 mm × 60 mm (Fig. 1a) and multiple lung tumors (Fig. 1b–d). We performed right radical nephrectomy, and subsequent pathological examination confirmed the diagnosis of clear cell RCC, G2>G3, INFa, v1, ly0, pT1b (Fig. 2a,b). His International mRCC Database Consortium risk was intermediate due to the anemia and the time from diagnosis to systemic therapy. Among the multiple lung tumors in chest CT images, the 35 mm lung hilar mass (Fig. 1d) was indistinguishable as either primary lung cancer or lung metastasis of RCC. A bronchoscopic biopsy failed to provide a pathological diagnosis due to the small amount of tissue collected. Furthermore, a mild lung interstitial opacity, which may progress to interstitial pneumonia induced by ICIs, was observed on chest CT. Therefore, we administered pazopanib 800 mg/day as the first‐line therapy for lung metastasis. The pazopanib dose was reduced to 400 mg/day because of hepatotoxicity. Subsequently, as hepatotoxicity worsened to grade 3, we discontinued pazopanib therapy. We switched to axitinib 5 mg twice daily as the second‐line therapy. During the pazopanib and axitinib therapies, the sizes of the lung metastases remained unchanged; only the lung hilar mass increased in size. Since the lung hilar mass was suspected to be a primary lung cancer, thoracoscopic lung lobectomy was performed. However, pathological examination of the resected mass revealed it was a lung metastasis of RCC (Fig. 2c,d). Due to failure of the previous therapies of pazopanib and axitinib, we decided to switch to ICIs. The mild interstitial opacity on the chest CT has remained unchanged for extended periods. A pulmonologist indicated that the interstitial pneumonia was inactive and that administering ICIs was acceptable. As a result, we used nivolumab as the third‐line therapy. After four cycles of nivolumab, CT showed PR for lung metastases. Simultaneously, the patient experienced blurry vision, and an eye examination by an ophthalmologist showed bilateral subretinal fluid, which is typically observed in acute VKH‐like uveitis (Fig. 3a,b). HLA typing test revealed that he had HLA‐DR4 (DRB1*04) alleles, which have been reported to be strongly associated with VKH.^4^ The patient was diagnosed with VKH‐like uveitis induced by nivolumab, and we therefore discontinued nivolumab treatment. He was started on topical treatment with subconjunctival steroid injection and steroid eye drops. His bilateral subretinal fluid was resolved and visual symptoms were improved (Fig. 3c,d), after which nivolumab was resumed while continuing the use of steroid eye drops. Since February 2021 through August 2024, following the initiation of nivolumab therapy, the patient has maintained PR and there has been no recurrence of VKH.

**Fig. 4:**
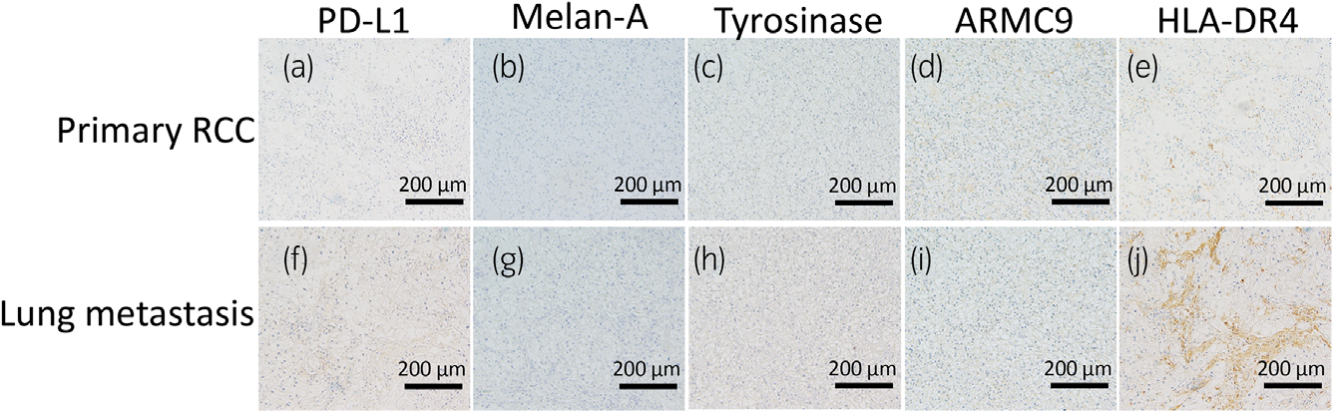
Immunohistochemical findings of primary RCC (a–e) and lung metastasis (f–j). The scale bar on all images is 200 μm. HLA‐DR4 expression is observed more strongly in the lung metastasis than in primary RCC (a, f: PD‐L1 immunostaining, b, g: Melan‐A immunostaining, c, h: tyrosinase immunostaining, d, i: ARMC9 immunostaining, e, j: HLA‐DR4 immunostaining).

We performed immunohistochemical staining on both the primary RCC and the lung metastasis to evaluate the expression of PD‐L1 and melanocyte proteins, including Melan‐A, tyrosinase, and ARMC9, which are candidate autoantigens of VKH, and HLA‐DR4. The expression of PD‐L1 and these melanocyte proteins was not detected in both primary RCC (Fig. 4a–d) and lung metastasis (Fig. 4f–i). However, we found that HLA‐DR4 expression was significantly higher in the lung metastases than in the primary RCC (Fig. 4e,j).

## Discussion

The patient developed VKH‐like uveitis, a rare irAE, while other irAEs were absent. Based on the Naranjo *et al*. Adverse Drug Reaction Probability Scale, ICIs are definitely associated with uveitis.^5^ The VKH‐like uveitis was managed by topical steroid therapy, and the patient maintained PR of lung metastases by resuming nivolumab therapy. We analyzed the expression of molecular targets relevant to immune reactions to elucidate the mechanism of VKH‐like uveitis development following nivolumab administration, as well as to explore the effectiveness of nivolumab for lung metastases. VKH is considered to be an autoimmune disease against melanocytes and closely associated with the HLA class II antigen, HLA‐DR4.^6^ This antigen is found in 88% of Japanese patients with VKH, whereas it is present in 41.7% of the general Japanese population.^3^, ^7^ HLA class II is present in APCs and triggers an immune response by presenting antigens to T cells.^7^ In VKH patients, melanocyte‐autoreactive T cells may get activated by HLA‐DR4 positive APCs.^4^ Nivolumab has the potential to enhance the activity of melanocyte‐autoreactive T cells by blocking the PD‐1/PD‐L1 signaling pathway.^8^ In the present case, the patient had HLA‐DR4 alleles, which can lead to VKH‐like uveitis induced by nivolumab.

Secondly, we discuss the factors contributing to the effectiveness of nivolumab in this case from immunohistochemical staining. In several carcinoma, many studies have reported the association between PD‐L1 expression and the effectiveness of ICIs.^9^ In the present case, we compared PD‐L1 expression levels in the primary RCC and the lung metastases, but PD‐L1 was not expressed in either. Given that the patient developed VKH‐like uveitis induced by nivolumab, we investigated whether the melanocyte antigens that trigger VKH were also expressed in the primary RCC and lung metastases. However, melanocyte proteins, including Melan‐A, tyrosinase, and ARMC9, which are candidate autoantigens of VKH, were not expressed in either. Furthermore, we compared HLA‐DR4 expression levels in the primary RCC and lung metastasis. We found that HLA‐DR4 expression was significantly higher in lung metastasis than in primary RCC. We hypothesize two reasons for the elevated HLA‐DR4 expression in lung metastasis: (1) the significant intratumoral heterogeneity identified in RCC^10^ suggests that only primary RCC tumors with high HLA‐DR4 expression could have metastasized to the lungs^11^; and (2) TKIs enhanced the expression of HLA‐DR as reported in other types of carcinoma.^12^ In the present case, it is possible that the administration of pazopanib and axitinib previous to nivolumab therapy may have elevated the HLA‐DR4 expression in the lung metastases. Specifically, we posited that elevated HLA‐DR4 expression in lung metastasis may enhance T‐cell activation, and this could increase the effectiveness of nivolumab for lung metastasis.

## Author contributions

Hitomi Imai: Conceptualization; data curation; formal analysis; investigation; methodology; resources; validation; visualization; writing – original draft; writing – review and editing. Tomoyuki Koguchi: Conceptualization; data curation; formal analysis; funding acquisition; investigation; methodology; project administration; resources; supervision; validation; visualization; writing – original draft; writing – review and editing. Yuki Harigane: Data curation; investigation; resources. Kei Yaginuma: Data curation; investigation; resources. Satoru Meguro: Data curation; investigation; resources. Seiji Hoshi: Data curation; funding acquisition; investigation; methodology; resources. Junya Hata: Data curation; funding acquisition; investigation; methodology; resources. Hidenori Akaihata: Formal analysis; funding acquisition; investigation; resources; writing – original draft; writing – review and editing. Soichiro Ogawa: Data curation; funding acquisition; investigation; methodology; resources. Yoshiyuki Kojima: Conceptualization; data curation; formal analysis; funding acquisition; methodology; project administration; resources; supervision; validation; visualization; writing – original draft; writing – review and editing.

## Conflict of interest

The authors declare no conflict of interest.

## Approval of the research protocol by an Institutional Reviewer Board

Not applicable.

## Informed consent

Not applicable.

## Registry and the Registration No. of the study/trial

Not applicable.
